# Increased hippocampal excitability in miR-324-null mice

**DOI:** 10.1038/s41598-021-89874-1

**Published:** 2021-05-17

**Authors:** Dan J. Hayman, Tamara Modebadze, Sarah Charlton, Kat Cheung, Jamie Soul, Hua Lin, Yao Hao, Colin G. Miles, Dimitra Tsompani, Robert M. Jackson, Michael D. Briggs, Katarzyna A. Piróg, Ian M. Clark, Matt J. Barter, Gavin J. Clowry, Fiona E. N. LeBeau, David A. Young

**Affiliations:** 1 Biosciences Institute, Newcastle University, Central Parkway, Newcastle upon Tyne, NE1 3BZ UK; 2 Bioinformatics Support Unit, Faculty of Medical Sciences, Newcastle University, Central Parkway, Newcastle upon Tyne, NE1 3BZ UK; 3 Orthopedics Department, First Hospital of Shanxi Medical University, Yingze District, Taiyuan, 030000 China; 4 Translational and Clinical Research Institute, Newcastle University, Central Parkway, Newcastle upon Tyne, NE1 3BZ UK; 5 School of Biological Sciences, University of East Anglia, Norwich, NR4 7TJ UK

**Keywords:** Neuroscience, Epigenetics in the nervous system

## Abstract

MicroRNAs are non-coding RNAs that act to downregulate the expression of target genes by translational repression and degradation of messenger RNA molecules. Individual microRNAs have the ability to specifically target a wide array of gene transcripts, therefore allowing each microRNA to play key roles in multiple biological pathways. miR-324 is a microRNA predicted to target thousands of RNA transcripts and is expressed far more highly in the brain than in any other tissue, suggesting that it may play a role in one or multiple neurological pathways. Here we present data from the first global miR-324-null mice, in which increased excitability and interictal discharges were identified in vitro in the hippocampus. RNA sequencing was used to identify differentially expressed genes in miR-324-null mice which may contribute to this increased hippocampal excitability, and 3′UTR luciferase assays and western blotting revealed that two of these, *Suox* and *Cd300lf*, are novel direct targets of miR-324. Characterisation of microRNAs that produce an effect on neurological activity, such as miR-324, and identification of the pathways they regulate will allow a better understanding of the processes involved in normal neurological function and in turn may present novel pharmaceutical targets in treating neurological disease.

## Introduction

MicroRNAs (miRNAs) are a class of single-stranded non-coding RNAs (ncRNAs) that modulate the expression of other RNA molecules. This regulation is achieved through utilising sequences within the 3′ untranslated region (3′UTR) of target RNAs which are complementary to the mature miRNA sequence in order to interact and consequently downregulate the translation of the target^1–3^. miRNAs have been predicted to affect the expression of vast numbers of transcripts throughout the genome^4,5^ via decapping and deadenylation of the target transcript^6–8^. Furthermore, the mechanism of gene regulation through miRNA activity is highly conserved across the plant and animal kingdoms^9–11^, underpinning the importance of these small ncRNAs. As such, it is unsurprising that miRNAs have been found to play major roles in many biological networks implicated in development and disease^12–17^.

miRNAs are crucial components in normal brain development and neurological function and a complete lack of miRNAs results in severe consequences. *Dicer1* encodes an essential endoribonuclease for miRNA processing and conditional *Dicer1* knockout models (KO) in individual neuronal populations present with severe neurological abnormalities^18–21^. These are most commonly due to upregulation of apoptotic pathways or downregulation of cell proliferation, resulting in reduced brain size. The impact of some individual miRNAs on brain development are so great that KO of the gene encoding only that miRNA can severely impact neurological functioning. For example, mice lacking miR-9, one of the most highly expressed miRNAs in vertebrate brains, present with severely abnormal telencephalic structures due to a lack of miR-9-mediated regulation of neural progenitor cell proliferation^22–24^. Double KO of genes encoding the miR-34b/c and miR-449 clusters, which are functionally redundant to one another^25^, mirrors *Dicer1* KO models in that apoptosis is upregulated, resulting in a reduction in basal forebrain size^26^. A related miRNA, miR-34a, is also important in normal brain function, and has been observed to produce an opposing effect to miR-34b/c in that it promotes apoptotic pathways when it is upregulated. Furthermore, miR-34a may contribute to the neuronal death that occurs in rodent models following seizures, as it has been shown to be upregulated following these events^27,28^. Individual miRNAs are therefore essential for normal brain development and in general these key neurological miRNAs exert their effects through shifting the balance between neurogenesis and apoptosis.

miR-324 has previously been suggested to function in the regulation of normal neural activity^29,30^ and is expressed more abundantly in the brain than in other tissues^31^. Experimentally validated target information for miR-324 is somewhat limited, but the two mature miR-324 arms, miR-324-5p and -3p, are each predicted to target and repress a large number of genes. Amongst the targets relevant for neurological activity, *App*, the gene encoding the amyloid precursor protein^32^, is one of the most confidently predicted^4,33^, potentially linking miR-324-5p to Alzheimer’s Disease^34^. miR-324-5p has also been investigated in relation to epilepsy; in a murine model of epilepsy, whereby wild-type (WT) mice were injected with pilocarpine in order to evoke seizure activity, the use of a miR-324-5p antagomir was found to reduce the incidence of epileptic events^30^, purportedly by downregulating the expression of *Kcnd2*, a gene encoding the potassium channel protein Kv4.2^29,30^. Deletion or mutation of *Kcnd2* has been shown to severely impair A-type K^+^ currents^35–37^. A detailed investigation into the cause of the association between miR-324 and epilepsy has however not yet been reported.

Our group has produced the first global miR-324-null mice, allowing high-throughput RNA sequencing (RNA-seq) to be applied in vivo for the first time to elucidate genes and pathways altered by a lack of *Mir324*, the gene encoding miR-324. Here, hippocampal slices from miR-324-null mice were found to display hyperexcitability in vitro, contrasting with what has previously been reported^30^. RNA-seq of hippocampus and neocortex identified genes that may be responsible for this phenotype and 3′UTR-luciferase assays were used to confirm direct interactions between miR-324 and putative targets identified using RNA-seq. Western blotting confirmed two of these putative targets, SUOX and CD300lf, were increased in miR-324-null hippocampal extracts. These interactions reveal novel regulatory activity of miR-324 on epilepsy-associated genes, allowing a better understanding of the processes involved in normal neurological function.

## Results

### Generation of miR-324 global knockout mice

**Figure 1:**
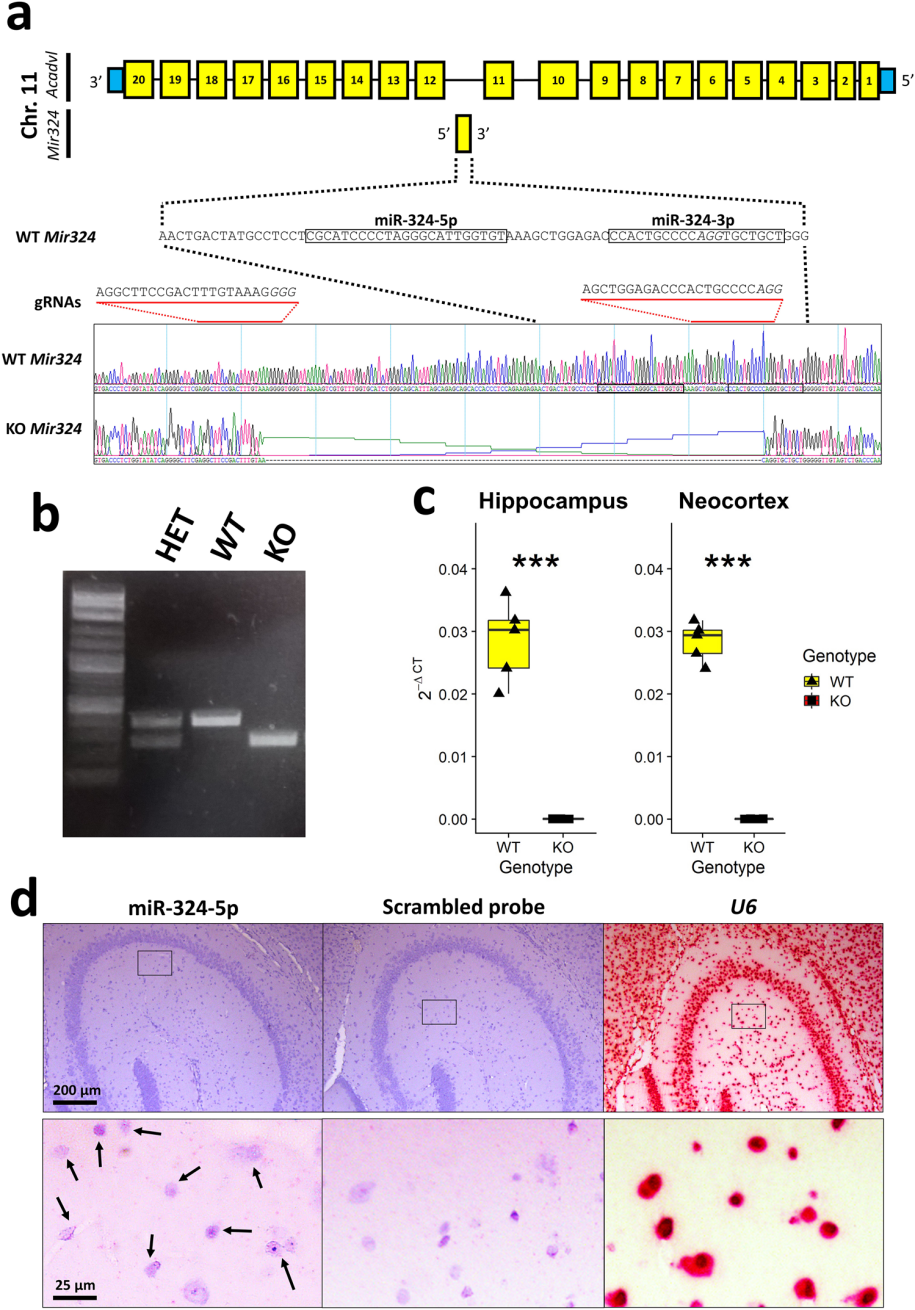
*Mir324* was knocked out in a C57/BL6 background in a 133 bp deletion. (**a**) The *Mir324* locus and surrounding region in relation to the knockout. The positions from which the mature miR-324-5p and -3p arms are encoded are indicated and text in italics shows the locations of the protospacer-adjacent motifs used for the knockout. (**b**) Routine breeding and genotyping by PCR was used to maintain the stock, and allowed easy identification of whether each individual was heterozygous (HET), wild-type (WT) or miR-324-null (KO) with respect to *Mir324*. (**c**) The expression levels of *Mir324* in hippocampus and neocortex tissue illustrate the lack of *Mir324* in 13.5 month old male miR-324-null (KO) mice relative to WT mice. *U6* was used as the control to normalise *Mir324* levels between samples. Here, *** signifies *p* value ≤ 0.001, where significance was calculated using two-sided Student’s *t*-tests. (**d**) miR-324-5p is shown to be expressed in the murine hippocampal CA3 region by in situ hybridisation. Arrows indicate the cells in which miR-324-5p expression is detected as punctate red spots. Hippocampal slices stained for negative and positive controls (a scrambled probe and *U6*, respectively) are also shown.

Since miR-324-5p is expressed more abundantly in the brain than in any other tissue^31^, we initially sought to determine the functional role of the microRNA using a knock-out mouse model. In order to assess the activity of miR-324-5p and -3p in mice, our group produced the first global knockout mouse model of miR-324 (Fig. 1a,b). The expression levels of *Mir324* in male 13.5 month old WT miR-324-null mice were measured using real-time reverse transcriptase polymerase chain reaction (qRT-PCR) in hippocampus and neocortex, confirming that miR-324-null mice completely lack expression of *Mir324* (Fig. 1c). Additionally, in situ hybridisation revealed that miR-324-5p is expressed across the murine hippocampus in WT mice, including the CA3 region (Fig. 1d). miR-324-null mice were born at the normal Mendelian ratio, were phenotypically normal with no obvious overt phenotype and survived with normal longevity.

### miR-324-null mice display electrophysiological abnormalities in the hippocampus

**Figure 2:**
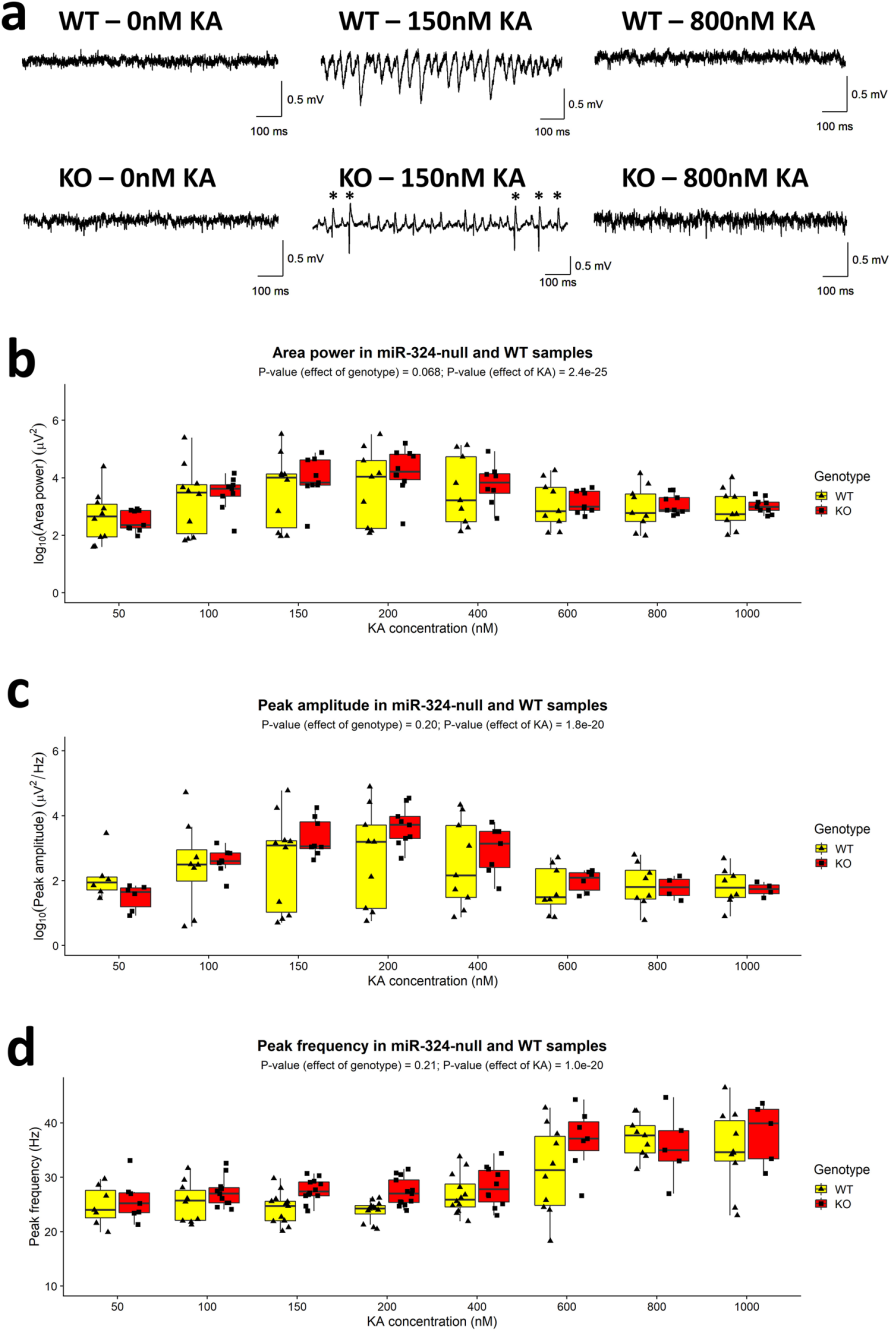
No change in gamma-frequency oscillations was observed between hippocampal slices taken from 5 month old female miR-324-null and WT mice (N = 16 slices obtained from 6 mice per genotype). (**a**) Example 1 s traces of LFP recorded from a single WT and miR-324-null slice in the absence of KA (baseline) and following bath application of KA at 150 nM and 800 nM. In both genotypes, no spontaneous oscillations were seen before KA application, but clear gamma-frequency activity was evident at 150 nM. In the miR-324-null slices, “spikes” in the LFP are indicated by *. Oscillations subsequently collapsed at higher KA concentrations (800 nM KA). (**b**–**d**) Comparisons between genotypes of area power, peak amplitude and peak frequency showed no significant differences, although area power was close to the 5% significance threshold (*p* value = 0.068). KA concentration significantly affected all three metrics. Slices that did not show clear gamma-frequency oscillatory activity at a particular concentration were omitted from analysis at that concentration, and therefore for some KA concentrations N < 16. *p* Values were calculated using 2-way mixed ANOVAs, although for peak amplitude and area power, the data was first log_10_ transformed as the initial data were not normally distributed.

In order to determine whether global miR-324-null mice exhibited any deficits in the generation of network activity, the local field potential (LFP) was recorded from the CA3 *stratum radiatum* in hippocampal slices obtained from 19 to 21 week old female miR-324-null and WT mice (16 slices obtained from 6 mice, for each genotype). Kainate (KA) was used to evoke gamma-frequency oscillatory activity (20–80 Hz) at a range of concentrations between 50 nM and 1 µM. At 0 nM KA, no spontaneous gamma-frequency oscillation was observed. As the KA concentration was increased, the power of the gamma-frequency oscillation increased in amplitude and area until 400–600 nM KA, at which point the oscillation collapsed as previously reported^38^. Hippocampal KA-evoked oscillatory activity was recorded at each concentration for 30 min and the final minute of each trace was used to measure area power, peak amplitude and peak frequency. All slices, both WT and miR-324-null, showed gamma-frequency oscillatory activity during the course of the experiment, but if no such activity was observed in a slice at a particular KA concentration then that slice was omitted from analysis at that concentration. Over the range of KA concentrations, no statistically significant differences were observed between genotypes for any of the metrics (Fig. 2), although area power showed a trend to be larger in the miR-324-null slices and was close to the significance threshold (*p* value = 0.068; N = 16 slices obtained from 6 mice per genotype, Supplementary Table S1). All three metrics were statistically significantly affected by the concentration of KA, independent of genotype (*p* values for area power, peak amplitude and peak frequency = 2.4 × 10^–25^, 1.8 × 10^–20^ and 1.0 × 10^–20^, respectively). With regard to peak frequency, a shift to a higher frequency is seen between 400 and 600 nM KA in miR-324-null slices, whereas in WT slices this shift is less consistent.

**Figure 3:**
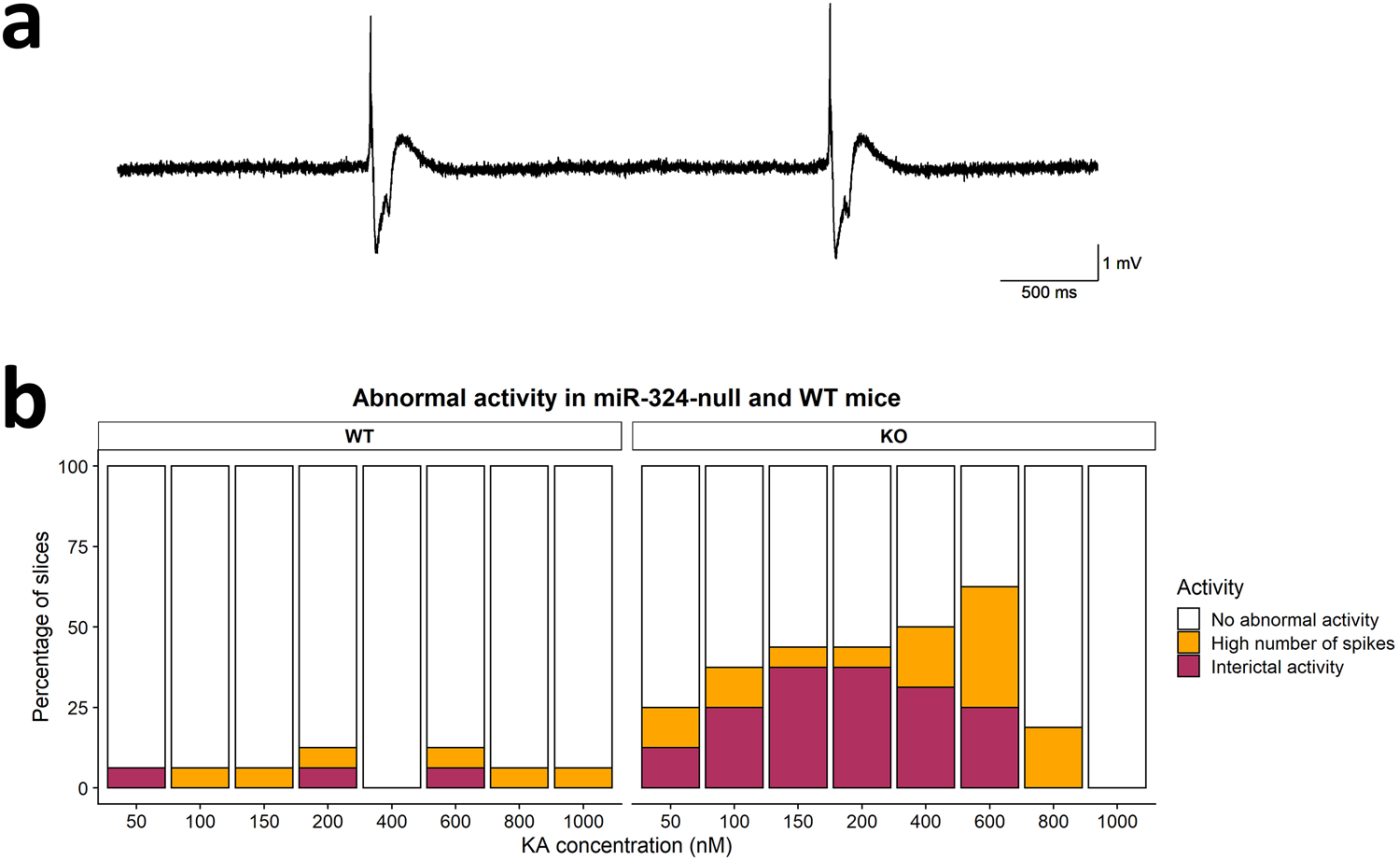
Increased excitability in hippocampal slices obtained from female 5 month old miR-324-null mice. (**a**) IIDs were observed in some slices from miR-324-null mice. (**b**) A far higher proportion of miR-324-null hippocampal slices showed IIDs or a high number of spikes than WT slices (N = 16 slices obtained from 6 mice per genotype). Any slice displaying at least one IID was classed as showing interictal activity, whilst slices showing no interictal activity but a high number of spikes were also grouped together. Slices were classed as showing high number of spikes if they had a greater number of spikes than the mean plus 2 SDs of spike number in the WT slices, for each KA concentration. For example, the mean plus 2 SDs of WT spikes at 150 nM KA was 15.0, and therefore any slices at 150 nM with more than 15 spikes were classed as having a high number of spikes. The most prominent distinction between genotypes is seen at 150 and 200 nM KA, at both of which 37.5% of miR-324-null slices displayed interictal activity, whereas the vast majority of WT slices at these concentrations showed no abnormal activity.

**Figure 4:**
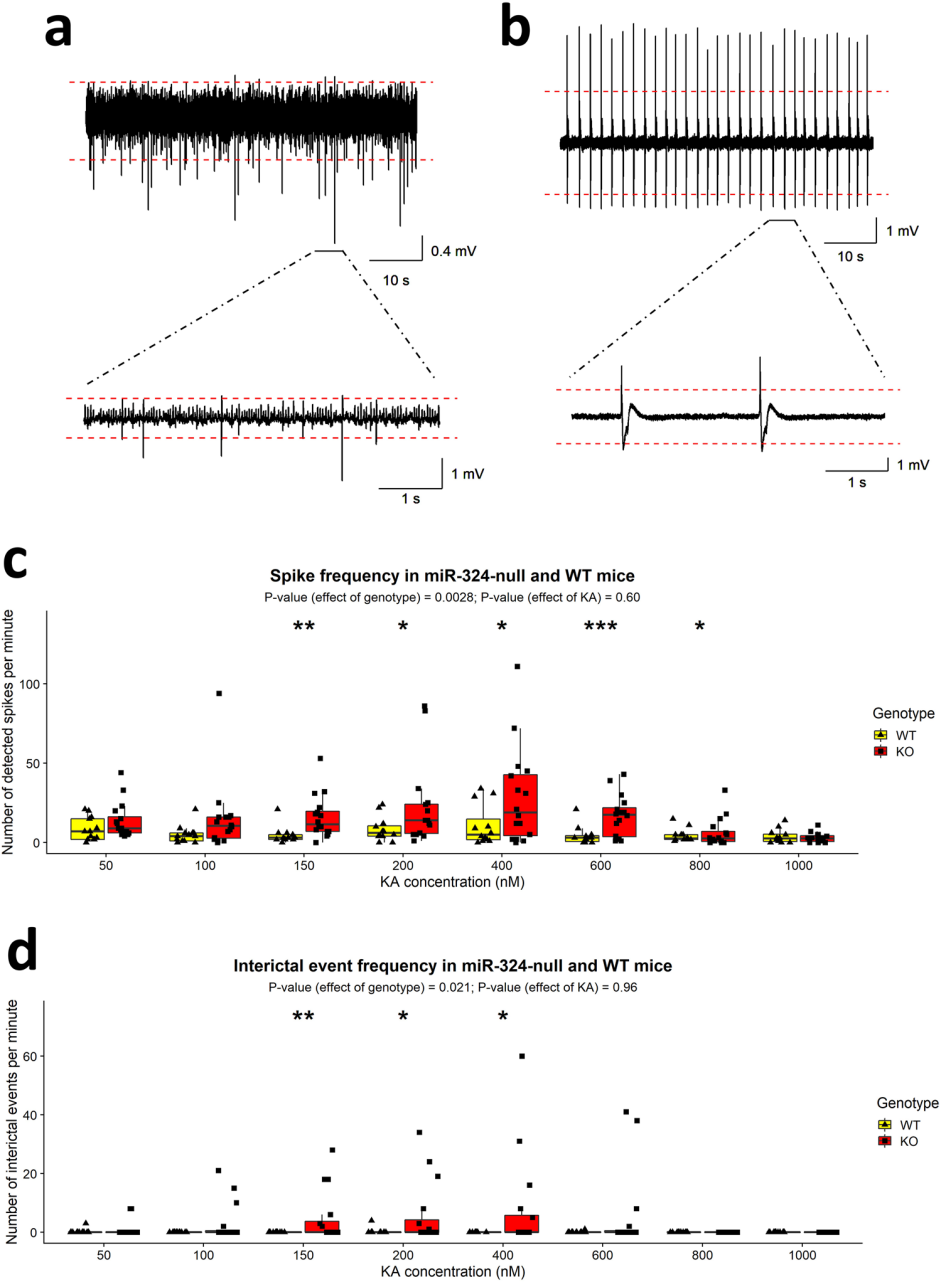
Comparison of measures of excitability in electrophysiology data measured in hippocampal slices taken from 19 to 21 week old female miR-324-null and WT mice (N = 16 slices obtained from 6 mice per genotype). (**a** and **b**) Examples of traces following bath application of 150 nM KA that show large numbers of spikes (peaks more than 5SDs from the baseline) and IIDs, respectively (both measured in miR-324-null slices). The red dashed lines indicate 5 standard deviations above and below the baseline. (**c** and **d**) Electrophysiological traces of miR-324-null mice contained a significantly higher frequency of spikes and IIDs than WT control traces. The majority of slices obtained from WT mice showed no IIDs and therefore the frequency for these slices is zero. *p* Values were calculated using 2-way mixed ANOVAs of ranks, as the initial data was not normally distributed. *, ** and *** indicate *p* values ≤ 0.05, 0.01 and 0.001 respectively for post-hoc testing using Tukey’s HSD tests.

Interestingly, in some hippocampal slices, particularly those obtained from miR-324-null mice, regular interictal discharges (IIDs) were evident following application of KA. In other slices, oscillations exhibited large so-called ‘spikes’ in the field potential recordings. Upon comparing the proportions of all slices exhibiting IIDs in miR-324-null and WT slices across the range of KA concentrations used, there was a noticeable increase in the number of slices showing IIDs in miR-324-null slices relative to WT slices, which was most apparent at 150 and 200 nM KA (Fig. 3). All IIDs observed in WT slices were obtained from a single mouse, whereas slices taken from 4 of the 6 miR-324-null mice produced IIDs after KA application. The number of detected spikes and IIDs per minute were both significantly increased in miR-324-null mice relative to WT control (*p* values = 2.8 × 10^–3^ and 0.021, respectively; N = 16 slices obtained from 6 mice per genotype), demonstrating that lack of *Mir324* increases the overall incidence of these hyperexcitable epilepsy-related events (Fig. 4). In particular, the frequency of spikes is significantly increased in miR-324-null slices between 150 and 800 nM of KA and the frequency of IIDs is significantly increased in miR-324-null slices between 150 and 400 nM of KA (*p* values ≤ 0.05, using Tukey’s HSD post-hoc tests). KA concentration was not found to significantly affect these metrics, independent of genotype.

### Hippocampal and neocortical RNA sequencing of miR-324-null mice

Both miR-324-5p and miR-324-3p are each predicted to target and repress a large number of genes; for both human and mouse, the miRNA target prediction algorithm TargetScan^4,33^ predicts over 3000 targets for each arm, although some of these overlap (Supplementary Figure S1a). There are reportedly two distinct isomiRs of miR-324-3p, miR-324-3p.1 and -3p.2^39–41^, which alter the predicted seed binding sequence and therefore also the predicted targets (Supplementary Figure S1b). However, most miRNA target prediction databases treat miR-324-3p.1 as the canonical form of miR-324-3p and hence this designation is used here. RNA-seq of RNA extracted from 13.5 month old male miR-324-null and WT mouse hippocampus and neocortex was undertaken in order to identify any predicted miR-324 targets or genes downstream of these in biological pathways that may be responsible for the observed epileptic phenotype (dataset freely available at GEO, accession GSE158337). The murine *Mir324* locus is within a predicted 3′UTR transcript variant of *Dvl2*, antisense to the *Acadvl* locus, but no reads from either genotype mapped to the predicted variant of *Dvl2* and reads that mapped to *Acadvl* did not differ between genotype (Supplementary Figure S2), confirming that hippocampal hyperexcitability identified was due to the removal of miR-324.

**Figure 5:**
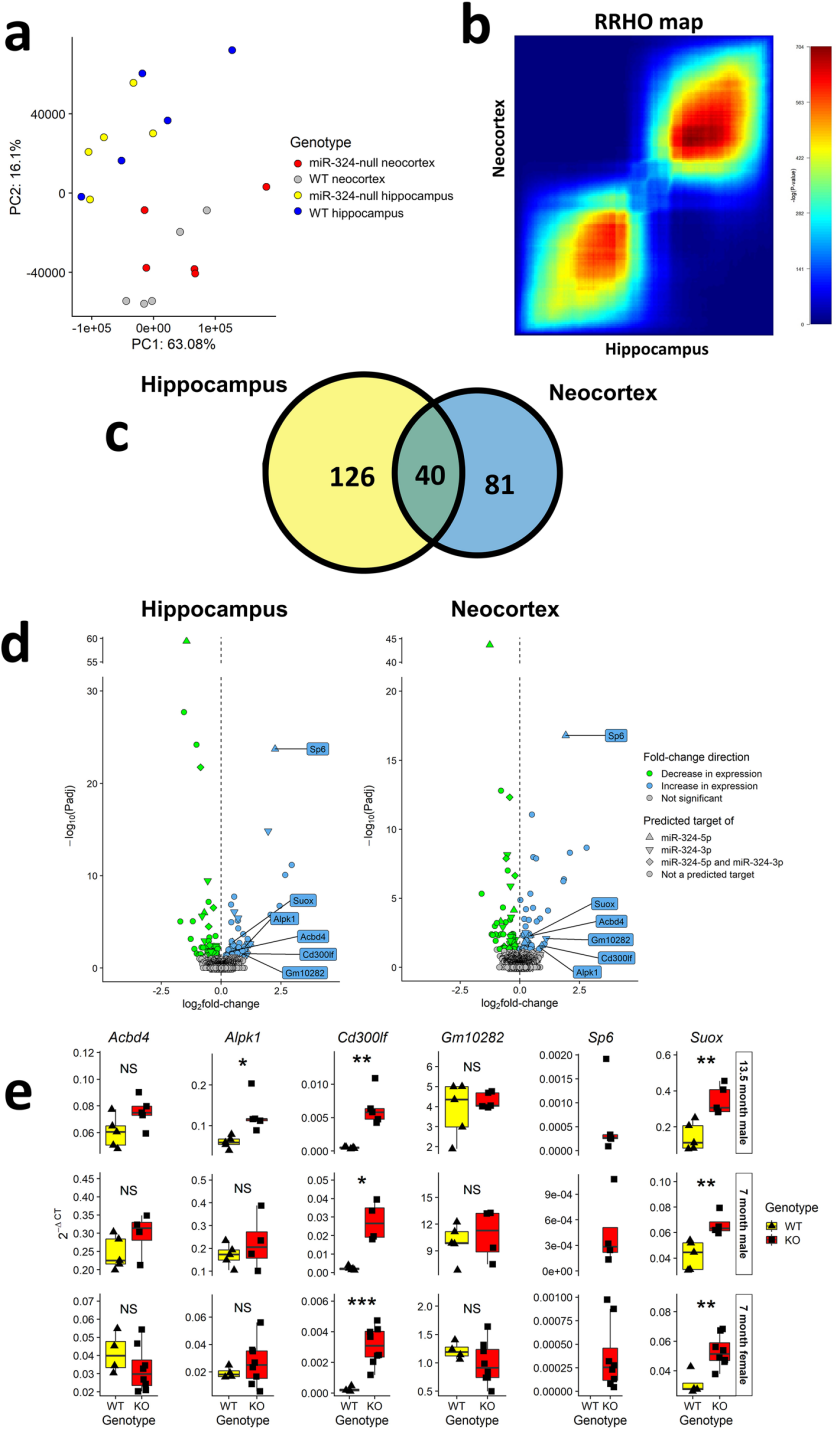
Initial analysis of RNA-seq of hippocampus and neocortex in 13.5 month old male miR-324-null and WT mice. (**a** and **b**) PCA analysis does not show as strong a segregation of samples by genotype as it does by tissue, although the results of hippocampus and neocortex RNA-seq experiments are well correlated using RRHO (sample permutation *p* value < 0.001). (**c**) Only 40 of the genes found to be DE in miR-324-null samples relative to WT controls were found to be DE in both tissues. (**d**) Volcano plots for hippocampus and neocortex tissue showing the fold-change, adjusted *p* value of each gene in addition to whether it is a predicted target of either arm of miR-324 according to the TargetScan algorithm^4,33^. Genes significantly increased in expression in miR-324-null samples are shown in blue, genes significantly decreased in expression are shown in green and genes showing no significant changes (*p* value > 0.05) are coloured grey. Genes found to be significantly upregulated in miR-324-null hippocampus and neocortex, in addition to being a miR-324 target, are labelled. (**e**) Of the genes that were predicted targets of miR-324 and significantly upregulated in both the hippocampus and neocortex RNA-seq, only *Cd300lf* and *Suox* were significantly DE in all cohorts by qRT-PCR analysis. *Sp6* was also highly upregulated, but it was detected in none of the WT samples in any cohort and so statistical significance could not be calculated. *, ** and *** indicate *p* values ≤ 0.05, 0.01 and 0.001 respectively, using two-sided Student’s *t*-tests to assess statistical significance.

Principal component analysis (PCA) of the RNA-seq data showed variance corresponding to the different brain regions to be greater than the variance corresponding to the genotypes, due to the relatively low number of differentially expressed (DE) genes (Fig. 5a). However, when comparing all genes ranked by fold-change the two datasets were highly correlative^42^ (Fig. 5b). The removal of the *Mir324* locus resulted in 126 genes significantly DE (adjusted *p* value ≤ 0.05) in the hippocampus and 81 genes significantly DE in the neocortex. A total of 40 genes were DE in both tissues (Fig. 5c; list of shared DE genes shown in Supplementary Table S2). A number of the DE genes were also predicted targets of miR-324-5p or canonical miR-324-3p according to the TargetScan miRNA target prediction algorithm^4,33^ (Fig. 5d). Of these predicted targets 6 were significantly upregulated (adjusted *p* value ≤ 0.05) in miR-324-null samples in both hippocampus and cortex (indicated in Supplementary Table S2). The expression of each of these genes was measured using qRT-PCR in the 13.5 month old male hippocampal samples and additionally in hippocampal samples from 7 month old male and female cohorts (Fig. 5e), and the expression *Cd300lf*, *Sp6* and *Suox* was confirmed to be consistently increased in miR-324-null samples.

**Figure 6:**
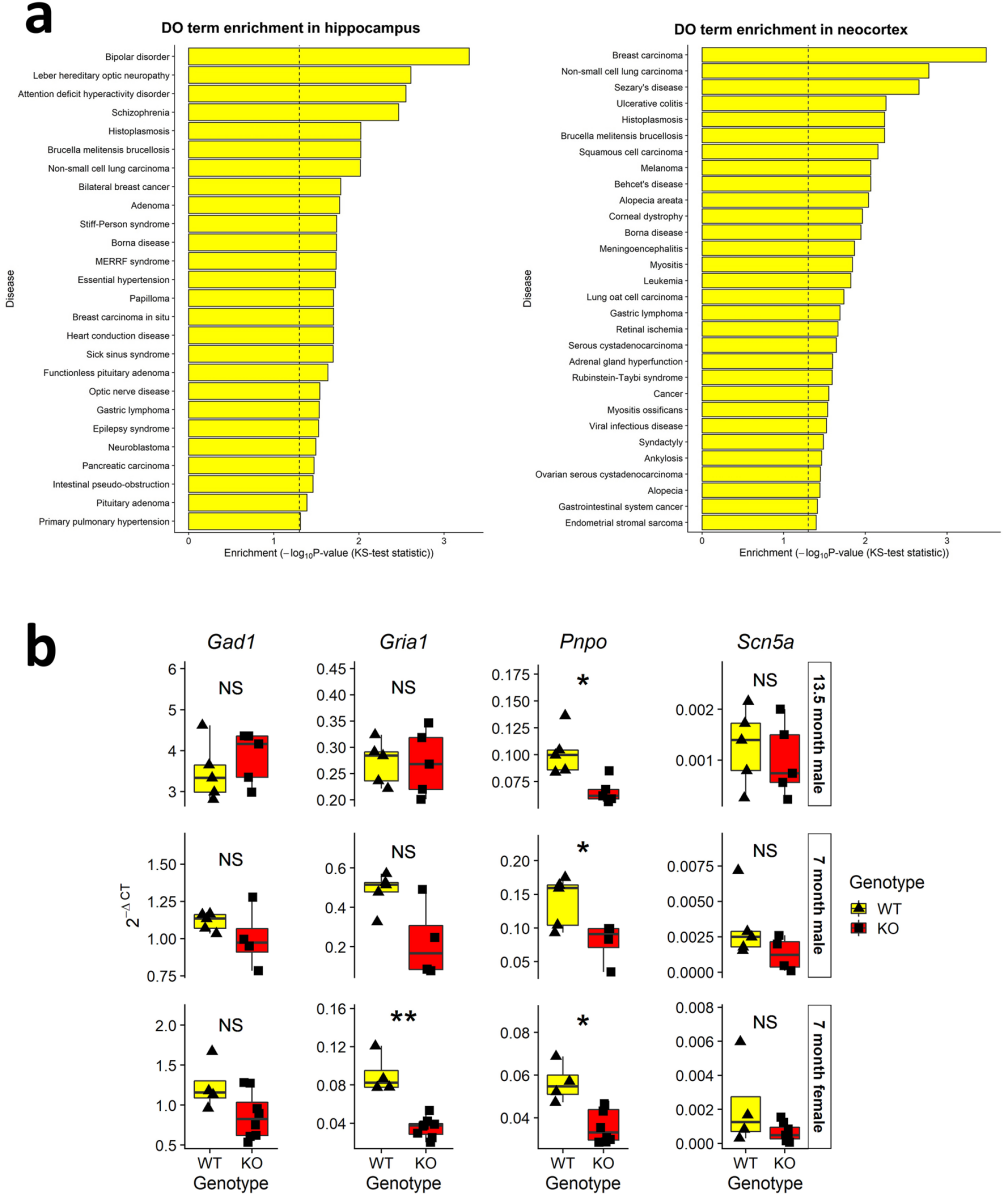
Relation of RNA-seq results to epilepsy. (**a**) Epilepsy syndrome was significantly enriched in the hippocampus RNA-seq, but not in the neocortex experiment (*p* values = 0.03 and 0.74 respectively), using terms and gene associations from the Disease Ontology. The significance of each Disease Ontology term was established using a Kolmogorov–Smirnov test. The dotted line on each plot shows where the cut-off for significance at a 5% level is found. (**b**) qRT-PCR quantification of the expression of epilepsy-associated genes was undertaken in the hippocampus tissue of the 13.5 month old male RNA-seq cohort and in 7 month old male and female hippocampal validation cohorts. * and ** indicate *p* values ≤ 0.05 and 0.01 respectively, using two-sided Student’s *t*-tests to assess statistical significance. Only *Pnpo* is significantly DE in all three cohorts.

Disease Ontology enrichment analysis^43^ showed that DE genes were significantly enriched for epilepsy syndrome in the hippocampus RNA-seq, but not in the neocortex (Fig. 6a). Therefore, related genes of interest (GOIs) were identified by filtering for epilepsy-associated genes in the Disease Ontology that were significantly DE in miR-324-null samples relative to WT controls, in either hippocampus or neocortex RNA-seq results (adjusted *p* value ≤ 0.05). Only 4 genes fitted these criteria, each of which was only significantly DE in the hippocampus (Table 1). These GOIs were measured using qRT-PCR in both the 13.5 month old (male) and the 7 month old (male and female) hippocampal samples, with only *Pnpo* identified as significantly DE in all three datasets (Fig. 6b). *Pnpo* was consistently downregulated in miR-324-null samples and additionally was not predicted as a target of either miR-324 arm. Therefore, it was not considered to be a candidate novel miR-324 target gene.

**Table 1:** Genes found to be DE (adjusted *p* value ≤ 0.05) in either the hippocampus or neocortex RNA-seq experiments that are associated with epilepsy according to the Disease Ontology^43^.

Gene	Hippocampus RNA-seq		Neocortex RNA-seq	
	logFC	Adjusted *p* value	logFC	Adjusted *p* value
*Pnpo*	− 0.523	3.20 × 10^–5^	− 0.253	0.248
*Scn5a*	1.21	0.00240	0.0309	0.989
*Gria1*	0.295	0.0282	0.0678	0.850
*Gad1*	0.270	0.0466	0.186	0.315

### Dual-luciferase assays reveal that *Cd300lf* and *Suox* are direct miR-324-5p targets

**Figure 7:**
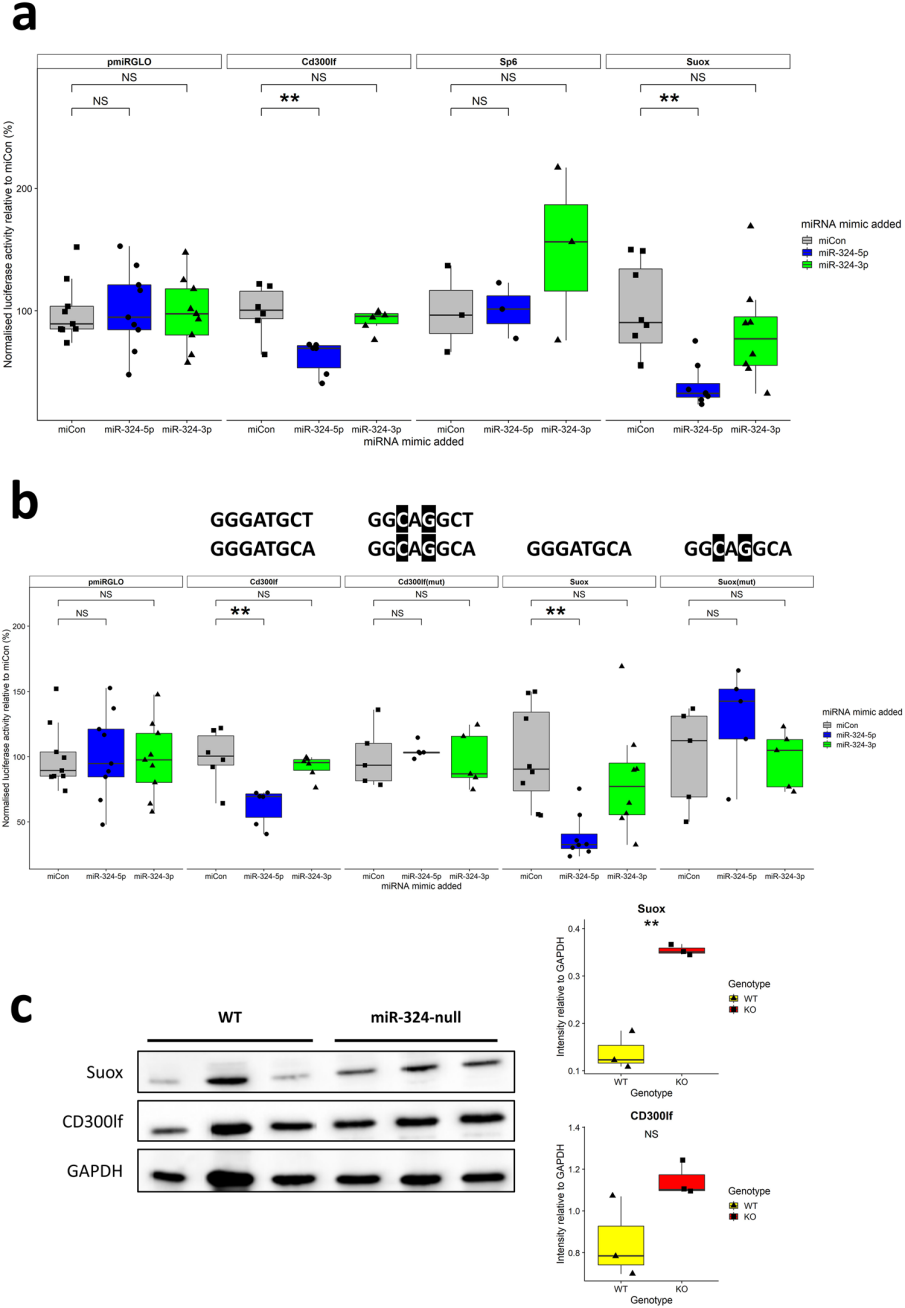
Identification of novel miR-324-5p targets using 3′UTR luciferase assays. (**a**) C3H10T1/2 murine cells were transfected with 3′UTR-pmiRGLO constructs and subsequently also with either a negative control miRNA mimic (miCon) or a mimic of miR-324-5p or miR-324-3p. Luciferase activity was measured after cells were incubated at 37 °C for 24 h. Relative luciferase activity was calculated as a ratio of renilla activity, an internal transfection control of pmiRGLO, and values were plotted as a percentage of mean miCon luciferase activity for each construct. *Suox* and *Cd300lf*, but not *Sp6*, were identified as direct miR-324-5p targets in vitro. (**b**) When 2nts of the miR-324 binding sites of the *Cd300lf* and *Suox* 3′UTRs were mutated, shown as *Cd300lf(mut)* and *Suox(mut)*, the miR-324-5p-mediated repression was inhibited in both cases. The predicted miR-324-5p binding sites of each 3′UTR are shown above the panel corresponding to that 3′UTR, with mutations highlighted in black. For panels **a** and **b**, the means of at least 3 independent experiments were used to test statistical significance in two-tailed Student’s *t*-tests. A negative control was included for each independent experiment (pmiRGLO) in addition to miCon and, after miR-324 targets were identified, one of these was used as a positive control for each independent experiment. (**c**) Western Blotting was used to confirm that the changes to the quantity of target gene mRNAs affected levels of the corresponding proteins in female hippocampal tissue aged 7 months. SUOX levels were significantly increased in miR-324-null samples relative to WT controls and although levels of CD300lf were not statistically significantly increased at a 5% level (*p* value = 0.077), there was a trend towards increased expression in miR-324-null samples. SUOX and CD300lf band intensities were normalised against GAPDH. In all panels, ** indicates *p* values ≤ 0.01.

Although *Cd300lf*, *Sp6* and *Suox* were validated to be significantly upregulated in miR-324-null mice at both 13.5 and 7 months of age and are predicted targets of miR-324, the interactions between miR-324 and the 3′UTRs of these genes were not necessarily direct. In order to test this, 3′UTR-luciferase reporter assays were undertaken in murine C3H10T1/2. Both *Cd300lf* and *Suox* showed statistically significant reductions in activity in the presence of miR-324-5p but not miR-324-3p or a miRNA negative control mimic (miCon; Fig. 7a). Additionally, when 2 nucleotides were mutated in the predicted binding sites of miR-324-5p in either of the *Cd300lf* and *Suox* 3′UTRs, the miR-324-5p-mediated downregulation was inhibited, strongly indicating that miR-324-5p binds the *Cd300lf* and *Suox* 3′UTRs in vitro and mediates repression of *Cd300lf* and *Suox* through these interactions (Fig. 7b). Western Blotting of SUOX and CD300lf from female hippocampal tissue aged 7 months revealed that the levels of both proteins increased in miR-324-null samples relative to WTs, although the increase in CD300lf levels did not achieve statistical significance at a 5% threshold (Fig. 7c).

## Discussion

The electrophysiological analyses undertaken here illustrated a clear hippocampal hyperexcitability with an increase in epilepsy-associated events in mice lacking *Mir324*. However, the networks regulating oscillatory activity in the hippocampi of these mice are evidently not severely impaired, as no significant changes were observed in the area power, peak amplitude or peak frequency in slices obtained from miR-324-null animals. It is therefore unclear whether mice lacking *Mir324* would develop any cognitive dysfunction relative to WT controls, and this should be a focus of future investigation. Additionally, it is unclear at what developmental timepoint the increased excitability emerges in the miR-324-null mice. In future studies it would be beneficial to undertake electrophysiology at earlier timepoints in these animals, in addition to developing a conditional KO model of miR-324, using for example the Fzd9-Cre mice, in which Cre recombinase expression is largely restricted the hippocampus and cortex^44,45^. This would allow analysis of the effect of miR-324 KO only in these specific tissues and additionally when miR-324 expression is removed at specific developmental timepoints, reducing the risk of any compensatory mechanisms.

Investigation into which specific neuronal populations have been affected in miR-324 null mice is also essential in order to assess whether the increased excitability is universal throughout the hippocampus or whether there are subregional differences. Furthermore, despite no evidence of any obvious seizure activity upon observation of the transgenic mice in the home cage or in response to handling, no detailed studies of behaviour were undertaken as part of this study. Therefore in order to elucidate both the causes and consequences of the increased IIDs in miR-324-null mice, in vivo recordings in awake animals, combined with behavioural assessments, are required.

The genetic cause of the increase in hyperexcitability is somewhat difficult to elucidate fully, but may stem from *Pnpo* and *Suox*, the two epilepsy-associated genes found to be significantly DE in the hippocampus, both in RNA-seq and qRT-PCR. *Pnpo* expression was decreased in hippocampal miR-324-null samples. In humans, mutant *PNPO* results in a specific form of epilepsy known as pyridoxal-phosphate dependent epilepsy (PPDE). This presents due to the inability of affected individuals to produce sufficient levels of pyridoxal-5′-phosphate (P5P), a key cofactor for many neurotransmitter biosynthesis reactions^46,47^. Patients affected by PPDE often present with neonatal epileptic encephalopathy, although some cases of later-onset epilepsy can also be effectively treated with P5P. This suggests that there may be a far broader spectrum of phenotypes for individuals lacking PNPO than is currently suggested in the literature^48,49^. It is, therefore, conceivable that the decreased expression of *Pnpo* in miR-324-null mice could play a role in the generation of increased epileptic events identified here.

*Suox* was also identified to be DE in miR-324-null mice, in addition to being a novel direct target of miR-324-5p in vitro. In humans, *SUOX* is associated with isolated sulfite oxidase deficiency (ISOD), a disorder caused by lack of functional SUOX. ISOD, like PPDE, is associated with increased epileptic events in patients^50^, although the relative gene expression of *SUOX* in ISOD is decreased, rather than the increased *Suox* expression identified in miR-324-null mice. It is possible that an excess of murine SUOX results in a phenotype similar to that of human SUOX deficiency, although there are no studies in the literature discussing this, and so exactly what effect an increase in *Suox* expression may have is currently unknown.

The second novel miR-324-5p target identified in this study, *Cd300lf*, has no direct link to epilepsy in the literature. However, it has previously been reported that in the THP-1 human cell line, treatment with CD300LF results in reduced levels of MMP-9^51^, a matrix metalloproteinase (MMP) which, along with the 22 other human MMPs, is key to the maintenance of extracellular matrix across many biological processes^52,53^. These include maintenance of the blood–brain barrier and homeostatic regulation of synaptic excitability, both of which have previously been demonstrated to be regulated in part by the activity of MMP-9^54–57^. Thus, the lack of *Cd300lf* repression in miR-324-null mice may result in reduced levels of MMP-9, therefore contributing to the abnormal neurological activity seen in vitro in hippocampal slices from miR-324-null mice. However, any CD300lf-mediated *Mmp9* repression would necessarily be subtle, considering we identified no significant change at a transcriptomic level. Further investigation is therefore required to identify whether this dysregulation of the CD300lf-MMP-9 pathway can affect MMP-9 levels to any biologically meaningful extent.

In addition to epilepsy, other neurological conditions identified in the Disease Ontology analysis included bipolar disorder, attention deficit hyperactivity disorder (ADHD) and schizophrenia. Interestingly, all these conditions have also been shown to be associated with cortical hyperexcitability^58–61^. We, therefore, cannot exclude the possibility that the increase in hippocampal excitability reported here could also point to a role of miR-324 in other neurological conditions.

Previously, downregulation of miR-324 using an antagomir has been associated with decreased epileptic activity^30^, compared to the increase in IIDs and spikes shown here. However, there were several notable differences between the two studies; Tiwari *et al*. (2019) used an injectable miR-324-5p antagomir to produce a transient miR-324-5p downregulation, and tested the effect of this in mouse models of epilepsy where pilocarpine had been used to induce *status epilepticus*. It is therefore possible that different phenotypes are seen because miR-324-5p was only transiently downregulated. Additionally, pilocarpine treatment in mice has been shown to induce changes in a large number of genes^62,63^, which may also play a role in the difference between the results identified in our study and those identified by Tiwari *et al*. Interestingly, the downregulation of *Kv4.2* by miR-324-5p observed by Gross *et al*. was observed only during or shortly after seizure activity and therefore future studies should explore whether miR-324 targets different genes and therefore produces different effects in the hippocampus during *status epilepticus* compared to under non-seizure circumstances.

Although the novel miR-324 targets identified and validated in this study appear to respond similarly to miR-324 in both murine tissue and the C3H10T1/2 cell line, it is possible that some targets of miR-324 were unable to be identified by the methodology we used. For example, *Gpc1* and *App*, the latter of which is one the most confidently predicted miR-324-5p targets^4,33^, were previously identified as direct targets of miR-324-5p using both transcriptomic and proteomic techniques in C3H10T1/2 cells^64^. However, in our RNA-seq experiment there is no significant increase in the expression of either gene. Another reported miR-324-5p target, *Kv4.2*, was identified using luciferase assays and proteomic analyses, but the authors reported that no changes in mRNA levels were identified^29^. We also identified no significant changes to *Kv4.2* mRNA levels between miR-324-null and WT mice using RNA-seq. All three of these genes have been implicated in neurological activity; *Gpc1* in relation to the developmental regulation of brain size^65^, *App* in the amyloidogenesis pathway^32^ and *Kv4.2* in the regulation of seizure activity and excitability^36,37^. Given the close relationship of these genes to the phenotypes observed in miR-324-null mice, all of these genes may well be also be direct miR-324-5p targets, and the fact that they were not identified in this study could be attributed to the lack of in-depth proteomic analyses in addition to the differences in models used. However, considering that the vast majority of protein level alteration can be attributed to a change in the amount of corresponding mRNA^66^, it is reasonable to assume that the target identification analysis undertaken in this study should have detected most direct miR-324 target genes that were expressed at a reasonable level in the murine hippocampus and neocortex. It should also be considered that in the case of *Gpc1* and *App*, *Mir324* was removed rather than overexpressed, and therefore the possibility remains that although these genes fulfil all in silico miR-324-5p target requirements, very little regulation by miR-324-5p occurs in vivo under physiological miR-324-5p levels.

The greatest limitations of the study presented here are the differences in age and sex between the mice used for each experiment. Although age and sex are always controlled for direct comparisons, there is a risk that the conclusions of each experiment do not necessarily hold for all timepoints, considering that there are notable increases in CA3 neuronal activity with age^67,68^ and many epilepsies are widely regarded as being more prevalent in either males or females^69^. Additionally it is possible that the expression of *Mir324* is altered with age or sex; many key miRNAs are upregulated in the ageing brain^70^, including miR-34a^71^, which also shows differential expression by sex^72^. The qRT-PCR validation of putative miR-324 target genes shows that, at least at a transcriptomic level, the novel miR-324 target genes identified in this study respond similarly to lack of miR-324 in samples from 13.5 month males, 7 month old females and 7 month old males, thus minimising these limitations, but not eliminating them entirely.

In summary, this study presents the first global miR-324-null mice and shows that hippocampal slices from these animals show increased excitability with an increased IID frequency relative to WT controls. Two epilepsy-associated genes are identified to be DE in the hippocampus and neocortex in mice lacking *Mir324*; *Suox* and *Pnpo*. Additionally, *Suox* is shown to be a direct target of miR-324-5p in vitro, as is *Cd300lf*, a gene indirectly associated with neurological function through MMPs. The abnormal hippocampal excitability seen in slices from miR-324-null mice may result from the altered expression of one or multiple of these genes. Further investigation into the downstream effects of *Mir324* removal may reveal novel pathways involved in ISOD and PPDE and therefore also may identify novel pharmaceutical targets for treating these conditions.

## Methods

### miR-324-null mouse models

All animal experiments were performed under licenses granted from the Home Office (United Kingdom) in accordance with the guidelines and regulations for the care and use of laboratory animals outlined by the Animals (Scientific Procedures) Act 1986 according to Directive 2010/63/EU of the European Parliament, and conducted according to protocols approved by the Animal Ethics Committee of Newcastle University and the Home Office, United Kingdom. Breeding and subsequent phenotyping was performed under licence P8A8B649A. All animal experiments were performed in compliance with the ARRIVE guidelines (http://www.nc3rs.org.uk/page.asp?id=1357). CRISPR/Cas9 guide RNAs (crRNAs) were designed using CHOPCHOP^73,74^. crRNAs linked with TRACR (sgRNA) were amplified by PCR with a pLKO vector (Addgene_52628) as template, with a T7 TRACR R primer (5′-AAAAGCACCGACTCGGTGCC-3′) in combination with a 5´ PCR primer that included a T7 sequence and specific crRNA (shown in bold; 5′-atgcatTTAATACGACTCACTATAgGG**AGGCTTCCGACTTTGTAAAG**GTTTTAGAGCTAGAAAT-3′; 5′-atgcatTTAATACGACTCACTATAgGG**AGCTGGAGACCCACTGCCCC**GTTTTAGAGCTAGAAAT-3′). This was converted to RNA using the MEGAshortscript T7 kit (Thermo Fisher Scientific). sgRNAs (50 ng/ml each) were mixed with recombinant Cas9 (ToolGen, CamBioScience Limited) and injected into the cytoplasm of donor mouse zygotes and transferred into recipient foster mothers, all essentially as previously described^75–77^. This resulted in a 133 bp deletion at the Mir324 locus. The mixed C57BL/6 and CBA/ca F_0_ mice were backcrossed onto C57BL/6 J and heterozygous animals crossed three times to eventually generate WT and miR-324-null lines. Genotype was confirmed by ear-notch PCR and Sanger sequencing (Forward primer: 5′-GTGCTGATCTACTCCTCCAACC-3′; reverse primer: 5′-AAATTCACAACTTTGGGGTGAT-3′). All experimentation after breeding and phenotyping was performed under licence P6EE54A50.

### miR-324-5p in situ hybridisation

Brains were obtained from 2 WT male mice (aged 4 months), fixed in 10% neutral-buffered formalin (w/v) and embedded in paraffin. A Microm HM355S Rotary Microtome (Thermo Fisher Scientific) was used to cut 5 µm horizontal sections at approximately − 5 mm along the DV axis relative to the bregma^78,79^. The levels of miR-324-5p (custom designed probe), *U6* (miRNAscope positive control) and a scrambled probe (miRNAscope negative control) were assessed in sections taken from each WT mouse using the miRNAscope HD Assay Red^80^ (Advanced Cell Diagnostics) following the manufacturer’s standard protocol. Samples were counterstained with Mayer’s Hematoxylin Solution (Sigma-Aldrich) and subsequently imaged using an Axiovert 200 Inverted Microscope (Zeiss).

### Hippocampus slice preparation

Female miR-324 KO and WT mice (aged 5 months) were anaesthetised by inhalation of 100% isoflurane, before intramuscular injections with ketamine (≥ 100 mg/kg) and xylazine (≥ 10 mg/kg). After the pedal withdrawal reflex had ceased, the mice underwent a transcardial perfusion with 30 ml of sucrose artificial cerebrospinal fluid (aCSF), via injection into the left ventricle. The sucrose aCSF was composed of the following: 204.5 mM sucrose, 3.0 mM KCl, 1.25 mM NaH_2_PO_4_, 2.0 mM MgSO_4_, 2.0 mM CaCl_2_·2H_2_O, 10.0 mM of D-glucose and 24.0 mM NaHCO_3_ (all from Sigma-Aldrich). The brain was excised and 450 µm horizontal hippocampal sections were cut using a vibratome (Model 5100 mz, Campden Instruments). The overlying cortex was trimmed from around the hippocampus, before being placed in a holding chamber at room temperature at an interface between carbogen gas and aCSF (with sucrose replaced by 126 mM NaCl) for 1 h. In order to avoid any confounding issues due to dorsal–ventral (DV) differences within the hippocampus^81–83^ we used only slices from the central portion of the hippocampus (between − 3.24 mm and − 5.04 mm along the DV axis relative to the bregma^78,79^).

### Electrophysiology data acquisition

Extracellular recording electrodes with resistances of 2–5 mΩ were produced from glass capillary tubes (Thin Wall Borosilicate with filament; Harvard Apparatus) using a micropipette puller (Model P-97, Sutter Instrument), before being filled with aCSF using a microfil syringe. Once the hippocampal slices had been in the holding chamber for approximately 40 min, they were transferred to a recording chamber that was kept at 30–32 °C, and through which 100 ml of aCSF was continuously recycled. The slices were left to acclimatise for 15 min, before LFPs were measured from the *stratum radiatum* layer of the CA3 region. The signal was amplified 10× by an AI-2010 differential amplifier (Axon Instruments) and background noise was minimised using a 50 Hz HumBug noise eliminator (Quest Scientific). Neurolog external filters (Digitimer Ltd.) were used to filter the signal at 1 Hz high-pass and 300 Hz low-pass, with a sampling rate of 5000 Hz, and an ITC-18 (NPI Electronic) was used to convert the analogue signal to digital. Spontaneous LFP activity was recorded for 30 min, before bath application of 50 nM KA (Sigma-Aldrich). The resulting activity was recorded for a further 30 min, before the concentration of KA was increased every 30 min to 100 nM, 150 nM, 200 nM, 400 nM, 600 nM, 800 nM and 1 µM. Trace recordings were performed using Axograph X v1.7.2 (Axograph Scientific).

### Electrophysiology data analysis

Power spectra were generated from the last minute at each KA concentration, using Axograph. The peak frequency, peak amplitude and power area (from 15 to 48 Hz) of oscillations from each trace were measured and compared between genotypes. For some slices at high KA concentrations, an additional faster frequency was observed in the power spectra in addition to the gamma peak. For the purposes of assessing differences in gamma band activity, only the lower gamma-frequency peak was measured. At each KA concentration, only slices showing clear gamma-frequency oscillations were included for analysis. The standard deviation (SD) of the amplitude for the final minute of each trace was also measured and Axograph was used to detect peaks in the LFP at least 5 SDs from the baseline of that trace. These were defined as “spikes” as they occurred during clear gamma activity and were not full IIDs. A proportion of events were identified manually as IIDs, which are established biomarkers of epilepsy and which result from imbalance in the excitation and inhibition in neural networks^84–86^. Additionally, in order to summarise the data, slices that showed any IIDs were classed as displaying interictal activity, and those showing no IIDs but a higher number of spikes than the mean plus 2 SDs of WT slice spikes at each KA concentration were classed as showing a high number of spikes. The remaining slices were classed as showing no abnormal activity in response to KA. Measurements were taken from 6 miR-324 KO mice and 6 WT mice, and multiple slices were taken from each of these mice (details shown in Supplementary Table S1), giving an overall sample size of 32, consisting of 16 WT slices and 16 KO slices. Each slice was treated as one sample, due to the considerable variability in the power of oscillations. For each metric, two-way mixed ANOVA tests or variants were used to assess statistical significance of the effect of genotype and KA concentration. Where the data was not normally distributed, it was either log_10_ transformed or a two-way mixed ANOVA of ranks was used.

### RNA extraction from murine tissue and cells

RNA was extracted from murine tissue using the *mir*Vana miRNA Isolation Kit (Thermo Fisher Scientific), following the manufacturer’s protocol to isolate total RNA. For RNA extraction from C3H10T1/2 cells, QIAzol Lysis Reagent (QIAGEN Ltd.) was used, following standard protocols. A NanoDrop™ 1000 Spectrophotometer (Thermo Fisher Scientific) was used to measure the RNA concentration and purity.

### RNA sequencing and analysis

Prior to RNA-seq, RNA samples were purified using the DNA-free DNA Removal Kit (Invitrogen) to remove any DNA contamination. The sequencing library was prepared using the Illumina TruSeq Stranded mRNA sample preparation kit following the manufacturer’s protocol and subsequent sequencing was performed on an Illumina NextSeq 500. Kallisto^87^ was used for pseudo-alignment and quantification, against the mouse GRCm38 (release 92) transcriptome^88^. Mapped transcripts were converted to a gene level using Tximport^89^ and DESeq2^90^ was used to calculate *p* values and log_2_ fold-changes (logFC), using the Benjamini–Hochberg method to adjust for multiple testing. Integrated Genomics Viewer^91,92^ was used to produce sashimi plots for each tissue, in order to analyse whether any splicing variation occurred upon removal of *Mir324*. Rank-rank hypogeometric overlap (RRHO)^93^ of the RNA-seq results from the hippocampus and neocortex was carried out using the RRHO package in R^42^, with the logFC of each gene used as the comparison statistic. Sample permutation *p* values were also computed using the RRHO package, calculated using the sum of the signal from genes sharing similar logFC values in both hippocampus and neocortex RNA-seq results. Putative miR-324 target genes were identified by filtering initial RNA-seq results for genes that were upregulated in miR-324-null mice in both hippocampus and neocortex tissue and that were predicted to be targets of either miR-324-5p or miR-324-3p by the TargetScan 7.2 algorithm^4,33^. Other GOIs from the RNA-seq results were identified by utilising the Disease Ontology^43^, using the term “epilepsy syndrome” to filter for relevant genes, with murine genes first converted to their human homologues using the BiomaRt R package^94^. The Disease Ontology was also used to test to test for enrichment in specific diseases, using a Kolmogorov–Smirnov test for significant enrichment with the GOseq R package^95^. For this, DE genes were defined as those with adjusted *p* values ≤ 0.05.

### Reverse transcription and quantitative PCR

Complementary DNA (cDNA) was reverse transcribed from total RNA using M-MLV Reverse Transcriptase (Thermo Fisher Scientific), following the manufacturer’s protocol for each GOI. cDNA was then quantified using TaqMan Fast Advanced Master Mix (Thermo Fisher Scientific). Reverse transcription of *Mir324* and the housekeeping gene *U6* were undertaken essentially as previously described^64^. GOI mRNA levels were quantified using a Quant Studio 3 (Thermo Fisher Scientific), using specific primers and Universal Probe Library (UPL; Roche Molecular Systems) probes (Supplementary Table S3) to measure the amplification of individual GOIs. Each sample was heated to 95 °C for 20 s, before undergoing 50 cycles of heating to 95 °C for 1 s and 60 °C for 20 s. The ΔCt method of normalisation was used to account for any differences between the samples, using *U6* to normalise *Mir324* levels and the housekeeping gene *Ndufa2* to normalise every other gene tested. Therefore, the normalised Cts (cycle thresholds) were calculated as follows: ${2}^{-({Ct}_{GOI}-{Ct}_{Normaliser})}$. The normalised Cts measured in miR-324 KO samples were compared against those measured in WT samples to assess whether there was any statistically significant difference for each GOI.

### Construction of 3′ untranslated region luciferase reporter plasmids

Fragments of the 3′UTR regions of putative miR-324 targets were amplified from cDNA, which was reverse transcribed from RNA extracted from C3H10T1/2 cells. PCR primers (Supplementary Table S4) were designed for In-Fusion HD cloning (Takara Bio Inc.) into the pmiRGLO luciferase reporter plasmid (Promega Corporation), which was digested with *Xho*I restriction endonuclease (New England Biolabs (UK) Ltd.). All predicted miR-324 binding sites were included in the amplified regions. Mutant *Suox* 3′UTR was synthesised as a gBlock (Integrated DNA Technologies), again designed for In-Fusion HD cloning. Mutant *Cd300lf* (at both predicted miR-324-5p binding sites) was produced by site-directed mutagenesis of the pmiRGLO-*Cd300lf* plasmid, using QuikChange Lightning (Agilent Technologies; mutagenesis sites shown in lower case; site 1 forward primer: 5′-AGGCAGGCTGCTTCAGGcAgGCTGTGTAAATCGTATC-3′; site 1 reverse primer: 5′-GATACGATTTACACAGCcTgCCTGAAGCAGCCTGCCT-3′; site 2 forward primer: 5′-AGCAGAAGGTGGAGGcAgGCAGAAGGAGTCAGG-3′; site 2 reverse primer: 5′-CCTGACTCCTTCTGCcTgCCTCCACCTTCTGCT-3′). Both mutant constructs contained mutations of 2 nucleotides within the predicted miR-324 binding sites. For all 3′UTR constructs except mutant *Cd300lf*, In-Fusion HD cloning was used, following the manufacturer’s protocol. All constructs underwent confirmation by Sanger sequencing (Source BioScience).

### Cell culture and dual-luciferase assay

Murine C3H10T1/2 cells were cultured at 37 °C and 5% (v/v) CO_2_ in Minimal Essential Medium (MEM), supplemented with 2 mM L-glutamine, 10% foetal bovine serum, 100 μg/ml streptomycin and 100 IU/ml penicillin, using vented T75cm^2^ flasks as previously described^64^. Cells were seeded onto 96 well plates at 5000 cells/well, before being incubated at 37 °C for 18 h. Subsequently, cells were transfected with the pmiRGLO-3′UTR constructs using FuGENE HD Transfection Reagent (Promega Corporation) following the manufacturer’s protocol. These cells were incubated at 37 °C for 4 h, media was aspirated, and the cells were transfected with miCon2 (Control miRNA Mimic (miCon)) or miR-324-5p or miR-324-3p mimics at a final concentration of 50 nM using DharmaFECT 1 (all Horizon Discovery) transfection reagent. The cells were incubated at 37 °C for 24 h after which the media was aspirated, cells washed with phosphate-buffered saline (PBS) and lysed using 30 µl of Passive Lysis Buffer (Promega Corporation). Luciferase level of each well was determined relative to an internal Renilla control using a GloMax-Multi Detection System (Promega Corporation) and reagents from the Dual-Luciferase Reporter Assay System (Promega). For each pmiRGLO-3′UTR construct, 6 technical replicates were measured for each of miR-324-5p, miR-324-3p and miCon. The mean values from at least 3 independent experiments were used to calculate statistical significance. A negative control was included in every independent experiment and, after miR-324 targets were identified, one of these was used as a positive control for each subsequent experiment.

### Protein extraction and Western Blotting

Murine hippocampal samples were ground into a fine powder on dry ice, before each sample was resuspended in 150 µl of a 1% (v/v) TritonX lysis buffer. Samples were subsequently kept on ice for 25 min, before undergoing centrifugation at 13,000×*g* for 10 min, cooled to 4 °C. The supernatant was quantified using a Bradford Assay and 10 µg was heated to 105 °C for 5 min to denature the protein, before being resolved on a 10% (w/v) SDS-PAGE gel and transferred to a PVDF membrane. The membrane was incubated overnight at 4 °C with the following primary antibodies: SUOX (Invitrogen, catalogue number PA5-21705; used at 1:200 dilution), CD300f./LMIR3 (R&D Systems, catalogue number AF2788; used at 0.2 µg/ml), GAPDH (Sigma-Aldrich, product number MAB374; used at 1:40,000 dilution). Band visualisation was undertaken using HRP-conjugated secondary antibodies (Dako, Agilent Technologies, product number P0448; used at 1:1000 dilution) and Immobilon Western Chemiluminescent HRP Substrate (Merck Millipore UK Ltd.) and subsequent quantification was performed using Fiji^96^.

### Statistical analysis

Where data was of a normal distribution (tested using the Shapiro–Wilk test for normality), statistical significance was assessed using Student’s two-tailed *t*-test for single comparisons or analysis of variance (ANOVA) for testing the effects of multiple variables on a continuous variable output; the test used for each comparison is indicated in the corresponding figure legend. Where data was non-parametric, Mann–Whitney U tests were used to assess significance of single comparisons and either log_10_ transformed two-way ANOVA tests or ANOVA of rank tests were used for the effects of multiple comparisons. Tukey’s HSD tests were used for post-hoc testing. All data analysis and statistical calculations were performed using R version 3.6.2^97^. Relevant R packages used for statistical analysis are cited.

## Supplementary Information

Supplementary Information.

## Data Availability

RNA-seq data is freely available at GEO (accession GSE158337) and all other datasets are available from the corresponding author on reasonable request.
