# Supplementary material for: Increased hippocampal excitability in miR-324-null mice
Source: Sci Rep. 2021 May 17;11:10452. doi: 10.1038/s41598-021-89874-1 (PMC8129095; doi:10.1038/s41598-021-89874-1)
Supplement: Supplementary file 1 — Supplementary Information. [file 41598_2021_89874_MOESM1_ESM.pdf]

Supplementary Data

Increased hippocampal excitability in miR-324-null mice

Dan J. Hayman[1], Tamara Modebadze[1], Sarah Charlton[1], Kat Cheung[2], Jamie Soul[1], Hua Lin[1], Yao Hao[1,3], Colin G. Miles[4], Dimitra Tsompani[1], Robert M. Jackson[1], Michael D. Briggs[1], Katarzyna A. Piróg[1], Ian M. Clark[5], Matt J. Barter[1], Gavin J. Clowry[1], Fiona E.N. LeBeau[1], David A. Young[1]*

[1]Biosciences Institute, Newcastle University, Central Parkway, Newcastle upon Tyne, NE1 3BZ, UK

[2]Bioinformatics Support Unit, Faculty of Medical Sciences, Newcastle University, Central Parkway, Newcastle upon Tyne, NE1 3BZ, UK

[3]Orthopedics Department, First Hospital of Shanxi Medical University, Yingze District, Taiyuan, 030000, China

[4]Translational and Clinical Research Institute, Newcastle University, Central Parkway, Newcastle upon Tyne, NE1 3BZ, UK

[5]School of Biological Sciences, University of East Anglia, Norwich, NR4 7TJ, UK

*To whom correspondence should be addressed. Tel: +44 191 2418831; FAX: Not applicable; Email: david.young@ncl.ac.uk

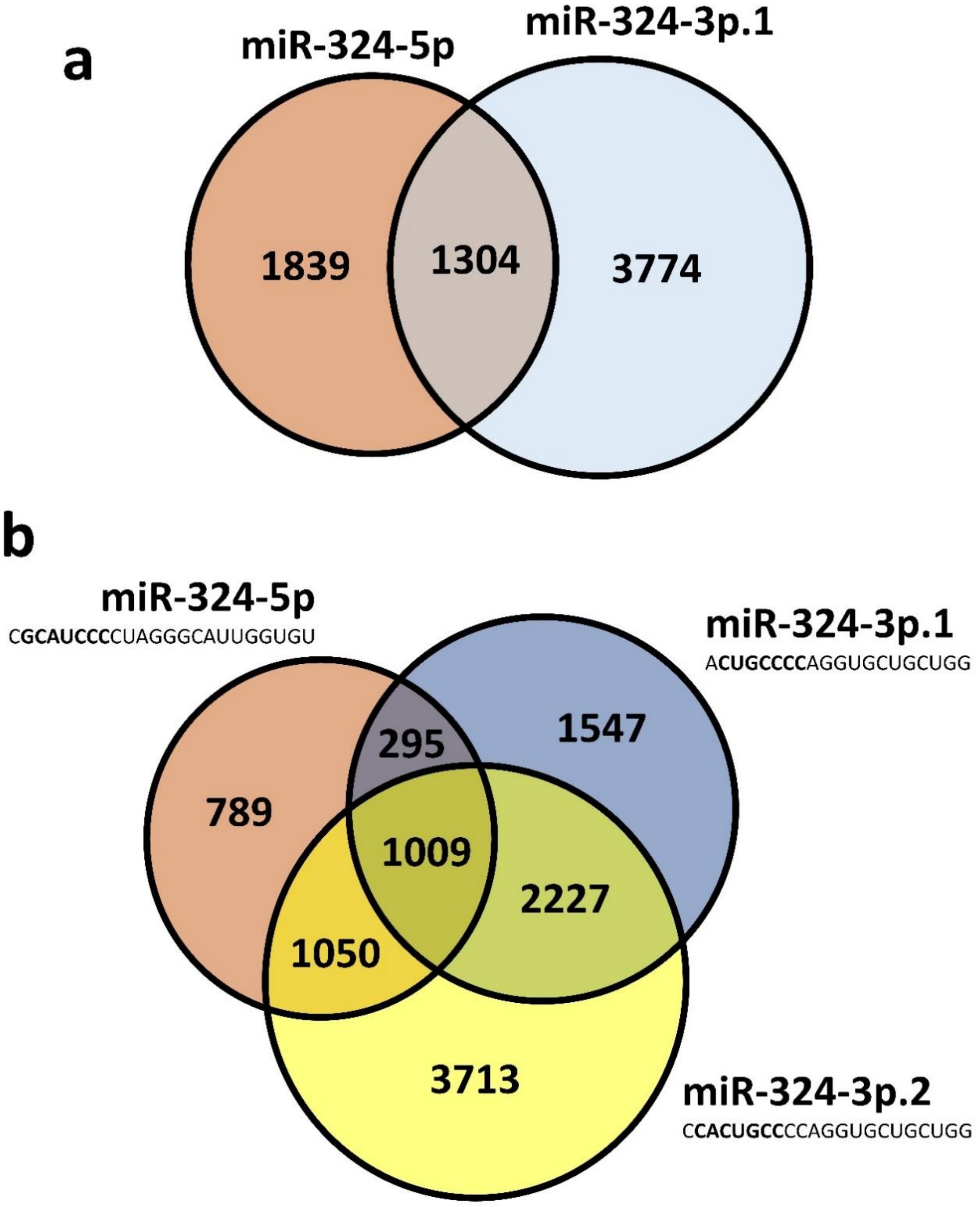

Supplementary Figure S1 - Numbers of predicted targets for each mature arm of miR-324, according to the TargetScan algorithm[4,33]. (**a**) Both miR-324-5p and canonical miR-324-3p are each predicted to target over 3000 mRNAs, although 1304 of these putative targets are shared between the two arms. (**b**) There are two distinct mature miR-324-3p sequences called miR-324-3p.1 and -3p.2. Generally, miR-324-3p.1 is used as the canonical isomiR, however a vastly different array of transcripts are predicted as miR-324-3p targets if miR-324-3p.2 is included in target prediction. The mature sequences of miR-324 are based on miRBase deep sequencing reads[40,41] and within these, the seed sequences are shown in bold.

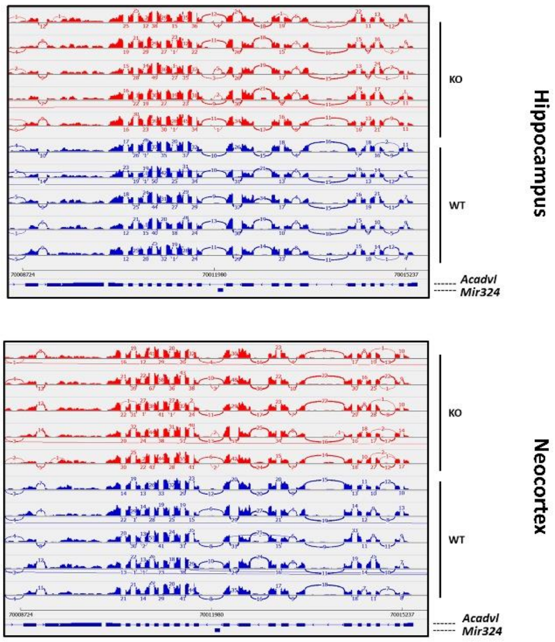

Supplementary Figure S2 - Very little splicing variation is seen in the region surrounding the *Mir324* locus between miR-324-null and WT samples, both in hippocampus and neocortex. Red indicates miR-324-null samples and blue indicates WT samples. The region shown is at the coordinates chr11:70,008,183-70,017,428.

Mouse	Number of hippocampal slices	Dorsal-Ventral slice coordinates
WT1	3	3, 4, 5
WT2	3	2, 3, 4
WT3	2	4, 5
WT4	3	3, 4, 5
WT5	2	4, 5
WT6	3	3, 4, 5
KO1	3	3, 4, 5
KO2	3	3, 4, 5
KO3	2	3, 4
KO4	3	3, 4, 5
KO5	3	4, 5, 5
KO6	2	4, 5

Supplementary Table S1 - Number of hippocampal slices obtained from each mouse and the corresponding coordinates of each hippocampal slice, using the Mouse Brain Atlas for Dorsal-Ventral position[81,82].

Gene	Hippocampus RNA-seq		Neocortex RNA-seq	
	logFC	Adjusted p-value	logFC	Adjusted p-value
Mrpl27	-1.45	3.84×10^{-60}	-1.26	1.99×10^{-44}
Sp6 *	2.25	1.81×10^{-24}	1.92	1.61×10^{-17}
Nme1	-1.03	6.26×10^{-25}	-0.786	1.59×10^{-13}
Gm4811	2.95	6.72×10^{-12}	2.80	2.17×10^{-9}
Phb	-0.567	3.54×10^{-10}	-0.531	7.00×10^{-9}
B230217C12Rik	-0.862	1.73×10^{-22}	-0.577	1.29×10^{-8}
Utp18	0.545	1.87×10^{-8}	0.564	1.04×10^{-8}
Mrpl10	0.421	1.32×10^{-7}	0.508	8.52×10^{-12}
Gm5540	2.44	1.85×10^{-7}	2.10	5.00×10^{-9}
Mllt6	-0.323	2.91×10^{-7}	-0.430	4.71×10^{-13}
Gm4875	2.68	8.15×10^{-11}	1.85	4.17×10^{-7}
Gm6304	2.07	1.68×10^{-6}	1.83	5.81×10^{-7}
Gm3555	-1.71	9.01×10^{-6}	-1.60	4.60×10^{-6}
Gm20390	0.428	1.12×10^{-5}	0.440	4.60×10^{-6}
Nme2	-1.55	1.98×10^{-28}	-0.705	4.71×10^{-5}
Gm10184	0.701	7.68×10^{-6}	0.559	4.88×10^{-5}
Dgke	-0.523	6.87×10^{-8}	-0.400	0.000355
Cbx8	0.762	0.00192	0.848	0.000296
Pigz	-1.18	8.79×10^{-6}	-0.904	0.00474
Cacng6	-1.27	0.000714	-1.18	0.00414
Chd9	-0.312	0.00533	-0.367	0.000236
Rapgefl1	-0.139	0.00594	-0.202	2.26×10^{-7}
Suox *	0.476	0.00165	0.447	0.00474
Gm5898	1.05	0.00641	1.18	7.71×10^{-5}
Rnf157	-0.307	0.00397	-0.309	0.00386
AC102745.1	-0.632	0.00594	-0.784	0.00396
Acbd4 *	0.356	0.0160	0.389	0.00544
Ncor2	-0.248	0.0217	-0.393	1.31×10^{-6}
Ece1	-0.217	0.00371	-0.201	0.0267
C1qb	-0.260	0.0275	-0.305	0.00484
Spop	-0.232	0.000350	-0.174	0.0347
Cep85	-0.307	0.0169	-0.303	0.0198
Hif1an	-0.245	0.0233	-0.265	0.0140
Ccl28	0.987	0.0186	1.04	0.0198
Gm10282 *	0.942	0.0311	1.10	0.00830
Eno1b	0.215	0.0384	0.245	0.00111
Alpk1 *	0.996	0.00481	0.842	0.0386
Cd300lf *	0.888	0.0275	0.916	0.0267
Smarce1	-0.402	0.0267	-0.397	0.0386
Mill2	-0.611	0.0380	-0.661	0.0305

Supplementary Table S2 - Genes significantly differentially expressed in both the hippocampal and neocortical RNA sequencing experiments in miR-324-null samples relative to wild-type controls, ordered by mean adjusted p-value. Genes statistically significantly upregulated in both hippocampus and neocortex, in addition to being predicted as a miR-324 target by the TargetScan algorithm[4,33], are indicated by *.

Gene	Forward primer	Reverse primer	UPL probe
Ndufa2	GAATGCTCGGAGGTGCAG	CTTTGCCGCTTAGCACATT	21
Acbd4	GCTGGCAGAGAGCTGACC	CCAACTCCTGTGGGCCTAT	7
Alpk1	AGAGGACAAGAGTGAGGACCAG	CGAAGGGCCACTTCATTTC	16
Cd300lf	TTGGAGGATGGTGAGGAGAC	TCCAGAGACTGTGCCTGCT	49
Gad1	ATACAACCTTTGGCTGCATGT	TTCCGGGACATGAGCAGT	27
Gria1	CCAATTTCCCCAACAATATCC	AAAGCTGTCGCTGATGTTCA	38
Pnpo	GGCCGAGAACTACTTCCATTC	GTTCCTCATTTTTCTTTCTCAGGT	71
Scn5a	GGATGAGGAGAACAGCCTTG	CACAACTTGGGATTCCTGCT	69
Sp6	GCAGCATCCTGCCTGTTC	GTCAAGGGTACCTCAGATGGA	10
Suox	GCCTCTGACCCTTATGCTGA	GGCGCTGGCTATTAATCCT	42

Supplementary Table S3 - Primers and UPL probes (Roche Molecular Systems) used to measure the mRNA levels of transcripts from specific GOIs.

3'UTR	Forward primer	Reverse primer	Product size (bp)
Cd300lf	**GCTCGCTAGCCTCGA**TGTACCTGCTTCCTTACCCG	**CGACTCTAGACTCGA**GCTCACGGAACAGAACAGTC	634
Sp6	**GCTCGCTAGCCTCGA**TGTCACATACCTGCCGTTCT	**CGACTCTAGACTCGA**AAGGACAGGCAGCTGAGAAA	2034
Suox	**GCTCGCTAGCCTCGA**TTGTACCCAAAGAACCACCTAG	**CGACTCTAGACTCGA**GGAAATCCCCTTGCATCCCA	581

Supplementary Table S4 - Primers used to amplify 3'UTR fragments from genomic DNA extracted from murine C3H10T1/2 cells. Sequences required for In-Fusion HD cloning (Takara Bio) are shown in bold.
